# Preoperative MRI-based radiomics analysis of intra- and peritumoral regions for predicting CD3 expression in early cervical cancer

**DOI:** 10.1038/s41598-025-12162-9

**Published:** 2025-07-23

**Authors:** Rui Zhang, Chunfan Jiang, Feng Li, Lin Li, Xiaomin Qin, Jiang Yang, Huabing Lv, Tao Ai, Lei Deng, Chencui Huang, Hui Xing, Feng Wu

**Affiliations:** 1 Department of Radiology, Xiangyang Central Hospital, Affiliated Hospital of Hubei University of Arts and Science, No. 136 Jinzhou Road, Xiangyang, 441021 Hubei Province People’s Republic of China; 2 Department of Pathology, Xiangyang Central Hospital, Affiliated Hospital Of Hubei University of Arts and Science, Xiangyang, Hubei People’s Republic of China; 3 Department of Obstetrics and Gynaecology, Xiangyang Central Hospital, Affiliated Hospital of Hubei University of Arts and Science, Xiangyang, Hubei People’s Republic of China; 4 Hubei Provincial Clinical Research Center for Cervical Lesions, Xiangyang, People’s Republic of China; 5 Institute of Gynecological and Obstetric Disease, Xiangyang Central Hospital, Affiliated Hospital of Hubei University of Arts and Science, Xiangyang, People’s Republic of China; 6 Department of Radiology, Tongji Hospital, Tongji Medical College, Huazhong University of Science and Technology, 1095 Jiefang Avenue, Qiaokou District, Wuhan, 430030 People’s Republic of China; 7 Department of Research Collaboration, R&D Center, Hangzhou Deepwise & League of PHD Technology Co., Ltd, No. 88 Longyuan Road, Cangqian Street, Yuhang District, Hangzhou City, Zhejiang Province 311101 People’s Republic of China

**Keywords:** Cancer imaging, Cancer microenvironment, Gynaecological cancer, Cancer

## Abstract

The study investigates the correlation between CD3 T-cell expression levels and cervical cancer (CC) while developing a magnetic resonance (MR) imaging-based radiomics model for preoperative prediction of CD3 T-cell expression levels. Prognostic correlations between CD3D, CD3E, and CD3G gene expressions and various cancers were analyzed using the Cancer Genome Atlas (TCGA) database. Protein–protein interaction (PPI) analysis via the STRING database identified associations between these genes and T lymphocyte activity. Gene Set Enrichment Analysis (GSEA) revealed immune pathway enrichment by categorizing genes based on CD3D expression levels. Correlations between immune checkpoint molecules and CD3 complex genes were also assessed. The study retrospectively included 202 patients with pathologically confirmed early-stage CC who underwent preoperative MRI, divided into training and test groups. Radiomic features were extracted from the whole-lesion tumor region of interest (ROI_tumor_) and from peritumoral regions with 3 mm and 5 mm margins (ROI_3mm_ and ROI_5mm_, respectively). Various machine learning algorithms, including Support Vector Machine (SVM), Logistic Regression, Random Forest, AdaBoost, and Decision Tree, were used to construct radiomics models based on different ROIs, and diagnostic performances were compared to identify the optimal approach. The best-performing algorithm was combined with intra- and peritumoral features and clinically relevant independent risk factors to develop a comprehensive predictive model. Analysis of the TCGA database demonstrated significant associations between CD3D, CD3E, and CD3G expressions and several cancers, including CC (*p* < 0.05). PPI analysis highlighted connections between these genes and T lymphocyte function, while GSEA indicated enrichment of immune-related pathways linked to CD3D. Immune checkpoint correlations showed positive associations with CD3 complex genes. Radiomics analysis selected 18 features from ROI_tumor_ and ROI_3mm_ across MRI sequences. The SVM algorithm achieved the highest predictive performance for CD3 T-cell expression status, with an area under the curve (AUC) of 0.93 in the training group and 0.92 in the test group. This MR-based radiomics model effectively predicts CD3 expression status in patients with early-stage CC, offering a non-invasive tool for preoperative assessment of CD3 expression, but its clinical utility needs further prospective validation.

**Supplementary Information:**

The online version contains supplementary material available at 10.1038/s41598-025-12162-9.

## Introduction

Cervical cancer (CC) is the most prevalent malignancy of the female reproductive system^1,2^. According to the World Health Organization (WHO), approximately 660,000 new cases and 350,000 deaths from CC occur globally each year^1^. In China alone, over 110,000 new diagnoses and 61,000 deaths are reported annually^2^. While traditional treatments such as surgery, radiotherapy, and chemotherapy are effective for early-stage CC, there is a growing need to explore innovative approaches that could further improve patient outcomes^3^. This highlights the urgent need for innovative clinical treatments. Immunotherapy, including immune checkpoint inhibitors (ICIs), antibody–drug conjugates (ADCs), and targeted therapies, has emerged as a promising approach for advanced and metastatic CC. However, patient responses to these therapies are highly variable^4,5^. Existing biomarkers such as PD-L1 and MSI-H have significant limitations in predicting immunotherapy outcomes^4^, underscoring the necessity of developing accurate predictive models to identify potential responders and optimize treatment strategies.

Recent studies increasingly emphasize the immunological roles of T cell subtypes, but their prognostic and therapeutic significance across different cancers remains insufficiently understood^6,7^. Histopathological evidence frequently demonstrates T lymphocyte infiltration within tumor nests, where these immune cells contribute to the tumor microenvironment and influence cancer progression and treatment outcomes^8,9^. Single-cell sequencing has revealed significant heterogeneity in T lymphocyte populations across malignancies, correlating with varying responses to radiotherapy, chemotherapy, and immunotherapy^8,10^. As a result, identifying reliable predictive biomarkers to enhance the efficacy of immunotherapy has become a critical focus in CC research.

At the molecular level, the CD3 complex (comprising CD3D, CD3E, and CD3G) is a pivotal marker for T lymphocyte identification and profiling, making it an essential target for immunological investigations^11,12^. This study utilized pan-cancer analysis of data from The Cancer Genome Atlas (TCGA) to evaluate the prognostic significance of T lymphocyte infiltration across various malignancies. Despite these advancements, objective and non-invasive methods for assessing T lymphocyte abundance in clinical settings remain unavailable.

Radiomics, a rapidly evolving field in medical imaging, presents a non-invasive and promising methodology for assessing the tumor immune microenvironment^13–15^. By extracting quantitative features from imaging modalities such as computed tomography (CT) and magnetic resonance imaging (MRI), radiomics enables phenotypic characterization of the immune landscape within tumors^14^. This approach deepens the understanding of tumor-immune interactions and supports the development of personalized therapeutic strategies with the potential to improve clinical outcomes^8^. Recent studies have increasingly investigated radiomics to identify associations between imaging features and tumor immune markers^11,12,14,16^. For example, CT-derived radiomic features have demonstrated potential in predicting CTLA4 expression in clear cell renal cell carcinoma^17^, while MRI-based radiomic features have shown efficacy in estimating CD3 + T lymphocyte levels in glioblastoma^18^.

Focusing on CC, where T lymphocyte infiltration significantly influences disease progression and therapeutic outcomes, this study leverages immunohistochemistry to detect CD3 as a marker of T cell presence in tumor tissues. A radiomics model was developed to identify imaging features indicative of CD3 expression levels, offering a non-invasive method to evaluate variations in T lymphocyte abundance. These radiomic features provide valuable insights into tumor immune status, aiding in the prediction of immunotherapy responses and enhancing prognostic assessments for patients with CC. In summary, this approach enables more personalized and effective treatment strategies.

## Materials and methods

### Data acquisition and comprehensive analysis of T lymphocytes in cancer

To investigate the clinical significance of T lymphocytes, CD3D, CD3E, and CD3G were selected as candidate gene markers. Normal tissue data from the GTEx database (https://gtexportal.org/) were integrated with tumor tissue data from The TCGA database (https://cancergenome.nih.gov) for a comprehensive joint analysis. Samples with zero gene expression values were excluded to mitigate potential biases arising from technical artifacts or non-expression states. RNA sequencing data underwent processing and standardization using the Toil pipeline, followed by log2 transformation (value + 1). Analyses included Kaplan–Meier (K-M) survival analysis, clinical parameter evaluation, immune checkpoint correlation analysis, and gene set enrichment analysis (GSEA), conducted via the Xiantao Academic platform with the R package (version 4.2.1; https://www.xiantaozi.com/).

The study analyzed 16 cancer types: bladder urothelial carcinoma (BLCA), breast invasive carcinoma (BRCA), cervical squamous cell carcinoma and endocervical adenocarcinoma (CESC), cholangiocarcinoma (CHOL), colon adenocarcinoma (COADREAD), esophageal carcinoma (ESCA), head and neck squamous cell carcinoma (HNSC), liver hepatocellular carcinoma (LIHC), lung adenocarcinoma and squamous cell carcinoma (LUADLUSC), ovarian serous cystadenocarcinoma (OV), pancreatic adenocarcinoma (PAAD), prostate adenocarcinoma (PRAD), rectum adenocarcinoma (READ), skin cutaneous melanoma (SKCM), stomach adenocarcinoma (STAD), and uterine corpus endometrial carcinoma (UCEC). Kaplan–Meier survival curves for these cancers were plotted, categorizing samples into high- and low-expression groups based on median gene expression levels to reduce subjective bias.

Protein–protein interaction (PPI) analysis utilized the STRING database (https://string-db.org/) with a confidence score threshold > 0.7. GSEA identified significant pathway differences between high- and low-expression groups, with criteria for significance set at a false discovery rate (FDR) < 0.25 and a *p*-value < 0.05.

Immunomonitoring correlation analysis examined the relationship between CD3D expression and immune checkpoint molecules, with results visualized in heatmaps to elucidate the immune landscape.

Immunohistochemistry (IHC) was performed using the Ventana Benchmark ULTRA automated staining system (Ventana Medical Systems, Tucson, AZ). Staining utilized a mouse monoclonal anti-CD3 antibody (clone 12,730, 1:100 dilution; Santa Cruz Biotechnology), with 3,3′-diaminobenzidine (DAB) as the chromogen. Positive controls confirmed strong CD3 staining in cervical carcinoma tissues, while phosphate-buffered saline (PBS) served as the negative control. Tissue images were scanned using the TEKsqray digital slide scanner (Shenzhen Shengqiang Technology Co., Ltd, China).

### Patients and clinical data

**Fig. 1:**
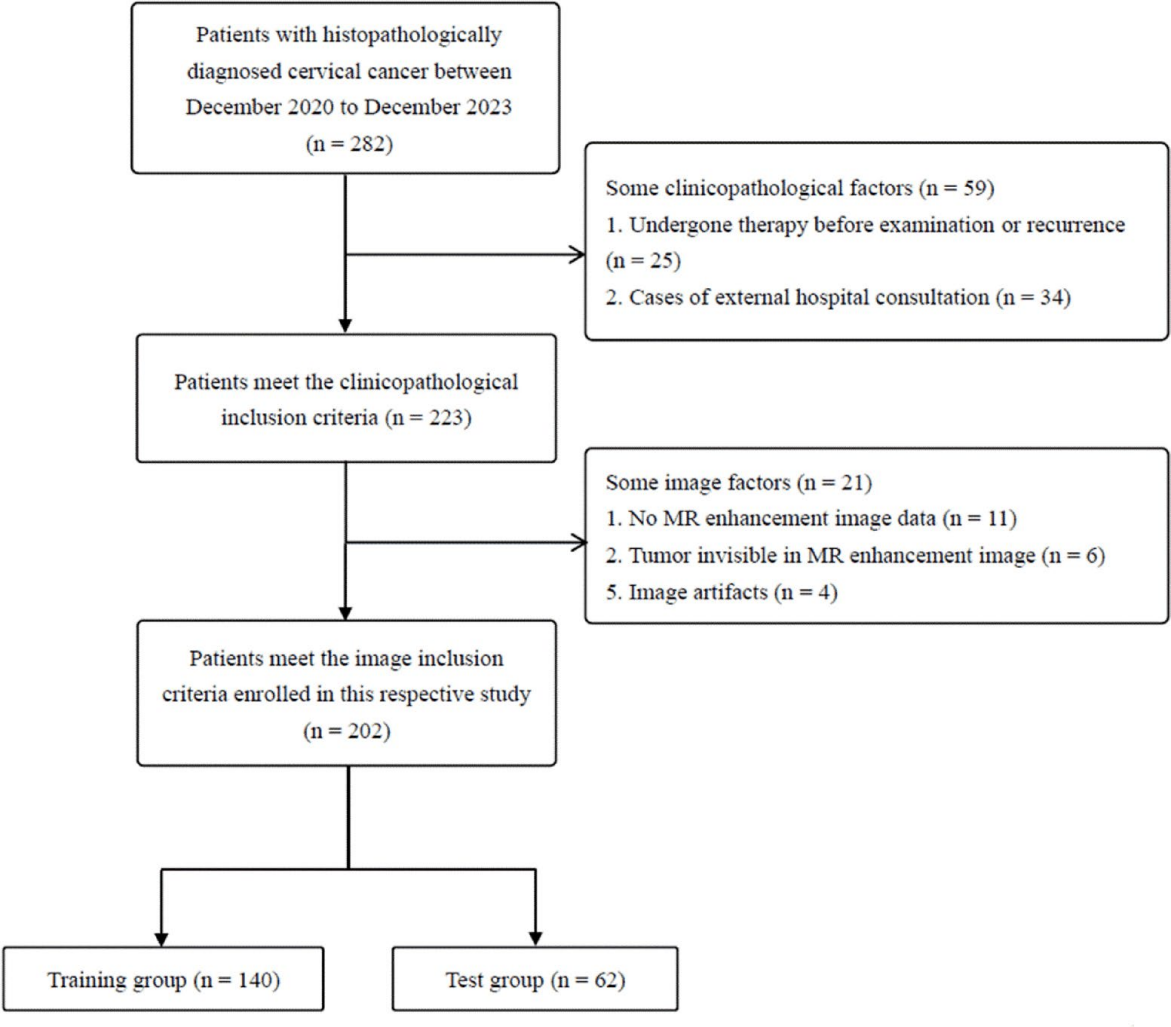
Flowchart depicting patient selection.

**Fig. 2:**
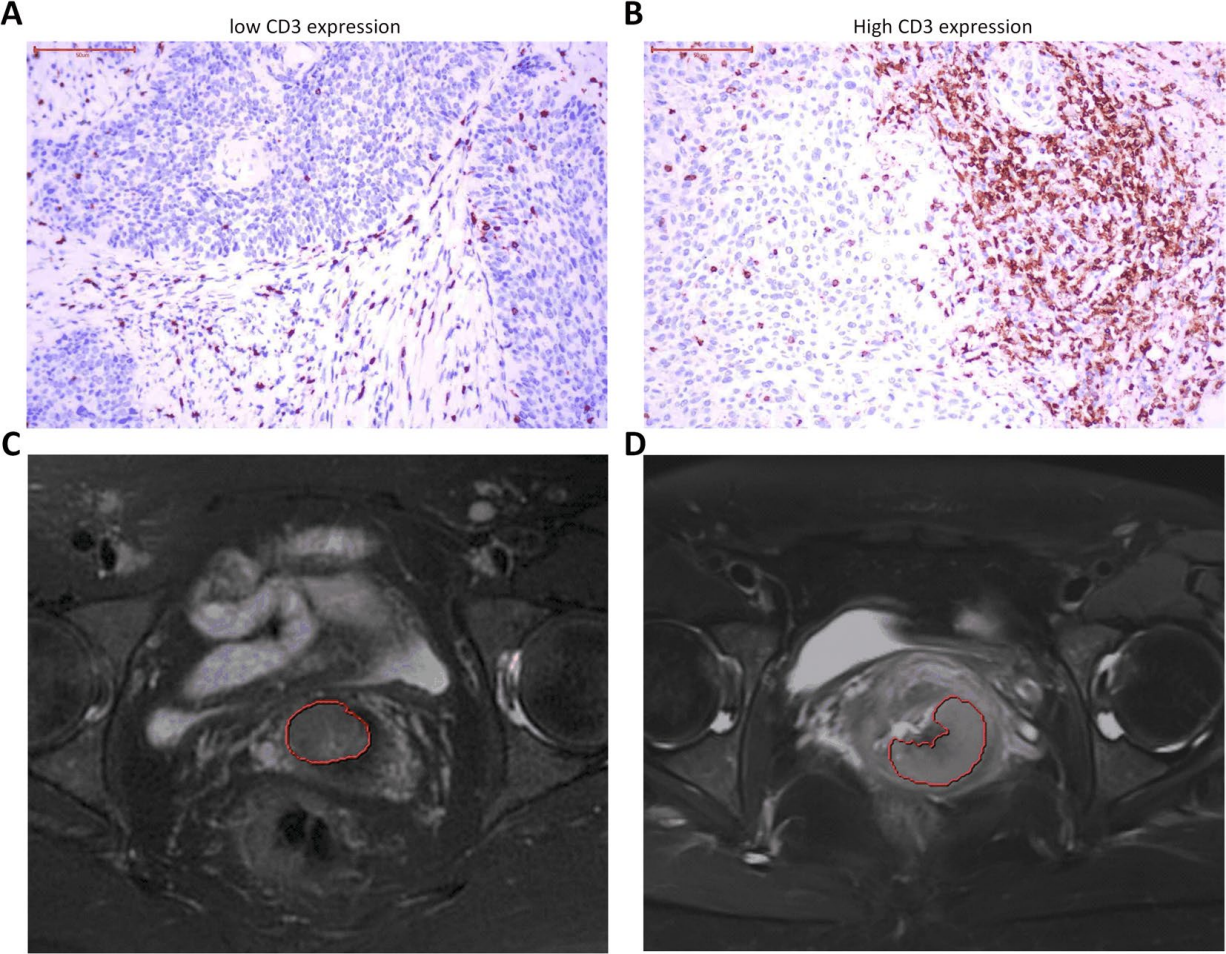
Immunohistochemistry and T2W imaging results from patients with high and low CD3 expression. Immunohistochemical results (scale bar: 50 µm) and the original image with a mask highlighting low CD3 expression are shown in (**A**) and (**C**), respectively; corresponding images with masks highlighting high CD3 expression are shown in (**B**) and (**D**).

This retrospective study received approval from the Institutional Review Board (IRB) of Xiangyang Centre Hospital, with informed consent waived due to its retrospective nature. All methods were performed in accordance with the relevant guidelines and regulations, including the Declaration of Helsinki. Patients with pathologically confirmed early-stage CC treated at the hospital between December 2020 and December 2023 were enrolled. Inclusion criteria comprised: (1) a diagnosis of early-stage CC (FIGO stages IA2-IIA) confirmed by surgical pathology, (2) availability of preoperative multi-parametric MRI (mpMRI) scans, (3) complete pathological data including CD3 expression status, and (4) no history of gynecological malignancies, prior pelvic surgeries, or previous radiotherapy or chemotherapy. Patients were excluded if they had undergone cervical conization, presented low-quality MRI images, or had lesions smaller than 5 mm in diameter on MRI. A total of 202 patients were included in the analysis, comprising 119 with low CD3 expression and 83 with high CD3 expression (Fig. 1). We observed that the FIGO stage and histological grade were correlated with the number of CD3 + T cells (Figure S1A,B). Based on this observation, we determined a cutoff value to distinguish CD3 expression levels, which was subsequently used as the classification criterion in our follow-up studies. High CD3 expression was defined as the presence of at least 20 CD3-positive cells per 1000 square micrometers of tissue (Fig. 2A, B).

Clinical and laboratory data were extracted from electronic medical records, encompassing CD3 expression status, patient age, clinical symptoms, obstetric history, blood tumor biomarkers, and peripheral blood counts.

### MRI acquisition and image analysis

All participants underwent mpMRI within 2 weeks prior to surgery, using a 3.0 T MRI scanner. The MRI protocol included axial T2-weighted imaging (T2WI) with fat suppression, diffusion-weighted imaging (DWI), and contrast-enhanced T1-weighted imaging (CE-MRI) sequences. Detailed scanning parameters for each sequence are listed in Table 1.

**Table 1:** MRI imaging parameters of each sequence in patients.

Sequence	Axial T2WI	Axial CE	Axial DWI
Technique	TSE	VIBE	EPI
TR/TE (ms)	2500/86	6.82/2.39	4300/60
Thickness (mm)	4	3	4
FOV (mm^2^)	260 × 260	320 × 320	260 × 260
Average	2	1	2
b-values (s/mm^2^)			50/800

T2WI, T2 weighted imaging; CE, contrast enhancement; DWI, diffusion weighted imaging; TSE, turbo spin echo; EPI, echo planar imaging; TR/TE, repetition time/ echo time; FOV, field of view; VIBE, volumetric interpolated breath-hold examination.

MRI images were independently reviewed by Radiologist A, an abdominal and pelvic imaging specialist with 5 years of experience. Radiologist A conducted the analysis blinded to clinical and histopathological data to minimize bias. For quantitative assessment, apparent diffusion coefficient (ADC) values were calculated using FireVoxel software (version 4.23; https://www.firevoxel.org/). Additionally, the presence of an intact dark stromal ring and the longest lesion diameter on axial T2WI images were recorded.

### Tumor segmentation, feature extraction, and selection

Tumor segmentation was conducted using the Deepwise Multimodal Research Platform (version 2.3, Beijing Deepwise & League of PHD Technology Co., Ltd, Beijing, China; https://keyan.deepwise.com), which enables precise three-dimensional (3D) delineation of tumor regions. Manual 3D regions of interest (ROIs) were delineated on DWI with a b-value of 800 s/mm^2^, T2WI, and CE-MRI images (Fig. 2C, D). The segmented ROIs from DWI were mapped onto corresponding ADC maps for consistency and analysis. Peritumoral regions with 3 mm (ROI_3mm_) and 5 mm (ROI_5mm_) radial thicknesses were automatically generated using the Deepwise platform to facilitate the analysis of peritumoral features.

Initial segmentation was performed by Radiologist A and subsequently reviewed and refined by Radiologist B, an expert with over 10 years of experience in gynecological oncology. To ensure the reproducibility of ROI segmentation, intra-observer agreement was assessed using the intra-class correlation coefficient (ICC). Thirty randomly selected patients underwent repeat segmentation after a 1-month interval, providing an objective measure of reliability and reproducibility.

To standardize datasets, all images were resampled to a uniform resolution of 1 × 1 × 1 mm using B-spline interpolation. Radiomic feature extraction was performed on the segmented regions using the Deepwise platform, encompassing first-order statistics, shape features, and texture features. Texture features included the gray-level co-occurrence matrix (GLCM), gray-level size zone matrix (GLSZM), gray-level run length matrix (GLRLM), and gray-level difference matrix (GLDM). Additionally, features were derived from preprocessed images using methods such as wavelet filtering, Laplacian of Gaussian (LoG) filtering, Gradient, LBP2D (Local Binary Pattern 2D), and nonlinear intensity transformations (e.g., square, square root, logarithm, exponential). A total of 1,502 radiomic features were extracted for each tumor volume, ROI_3mm_, and ROI_5mm_ across ADC, T2WI, and CE-MRI sequences. Detailed categorization and statistics of radiomic features are provided in Supplementary Table S1.

To ensure the reliability and effectiveness of our radiomics features, we implemented a rigorous three-step feature selection process. Initially, the Intraclass Correlation Coefficient (ICC) with a threshold of > 0.8 was employed to evaluate the reproducibility of features across repeated measurements or observers. Features with high ICC values were retained to ensure robustness and reliability, consistent with best practices in radiomics research. Subsequently, we performed a pairwise Pearson correlation analysis to eliminate highly collinear features (threshold: |r|> 0.8), which helps to reduce feature redundancy and mitigate multicollinearity effects during model training. Finally, the F-test (ANOVA) served as a univariate filter method to assess the discriminatory capacity of each feature in distinguishing outcome classes, preserving features exhibiting significant statistical relevance for classification purposes.

### Model construction and evaluation

Predictive models were constructed using five established machine learning algorithms: Support Vector Machine (SVM), Logistic Regression, Random Forest, AdaBoost, and Decision Tree. These models were trained on features extracted from multiple mpMRI sequences, including CE-MRI, T2WI, and ADC images. Given the high dimensionality and complexity of medical imaging data, coupled with the risk of overfitting, SVM was selected as the final algorithm due to its superior performance and robustness.

We performed hyperparameter tuning using grid search on the training set for all five machine learning models evaluated in this study. The best-performing hyperparameter combination for each algorithm was selected based on performance on test group, rather than k-fold cross-validation. And default settings were not used for any of the models. Details of the hyperparameter optimization strategy for five machine learning models are provided in Supplementary Table S2.

To explore the impact of tumor heterogeneity and the peritumoral microenvironment, models were developed using tumor ROIs and varying peritumoral ROIs, specifically the tumor ROI (ROI_tumor_) and peritumoral rings at 3 mm (ROI_3mm_) and 5 mm (ROI_5mm_) from the tumor boundary. This approach sought to determine the optimal peritumoral ROI for predictive modeling (Supplementary Table S3).

A clinical model was also constructed by incorporating significant clinical risk factors derived from clinical, laboratory, and conventional imaging characteristics. The top-performing radiomics model was subsequently integrated with the clinical model to form a comprehensive predictive model designed to enhance overall performance. In the radiomics analysis, considering the definition of high CD3 expression, the distribution of FIGO subgroups was regarded as a potential confounding factor. We constructed a distribution table for high and low CD3 expression groups across FIGO IA, IB, and IIA subgroups and evaluated the differences between groups using the chi-square test. Additionally, we explored the impact of incorporating FIGO staging as a variable into the clinical model and compared it with the model without this variable using the Delong test to assess the differences between them.

Model discrimination was evaluated through receiver operating characteristic (ROC) curve analysis, with metrics including area under the curve (AUC), accuracy, sensitivity, specificity, positive predictive value (PPV), and negative predictive value (NPV). Calibration and decision utility were assessed using calibration curves and Decision Curve Analysis (DCA) for both training and test cohorts. Additionally, SHapley Additive exPlanations (SHAP) values were employed to interpret the contribution of individual features to predictions, providing transparency into the model’s decision-making process.

### Statistical analysis

Statistical analyses were conducted using the Deepwise Multimodal Research Platform (version 2.3) and Xiantao Academic website with the R package (version 4.2.1; https://www.xiantaozi.com/). Continuous variables were tested for normality and variance homogeneity, with comparisons made using the Student’s t-test or the Wilcoxon rank-sum test, as appropriate. Categorical variables were compared using the Chi-square test or Fisher’s exact test.

Survival analysis was performed using the Kaplan–Meier method, with log-rank tests for comparing survival curves between groups. The “survival” (version 3.3.1), “survminer” (version 0.4.9), and “ggplot2” (version 3.3.6) R packages were utilized for visualization. PPI analysis was conducted using the STRING database with a confidence score threshold > 0.7 to ensure reliability. GSEA compared CD3D high- and low-expression groups using DESeq2 and the “clusterProfiler” package, identifying significant pathways (FDR < 0.25) and visualizing results with “ggplot2.”

The relationship between CD3D expression and immune checkpoint molecules was assessed via Spearman correlation analysis, with results visualized as a heatmap using “ggplot2.” Statistical significance was set at a *p*-value < 0.05. All data preprocessing steps were performed in accordance with CLEAR (Checklist for Artificial Intelligence in Medical Imaging) guidelines. The CLEAR checklist is provided in Supplementary 2.

## Results

### Bioinformatics analysis in the TCGA database

**Fig. 3:**
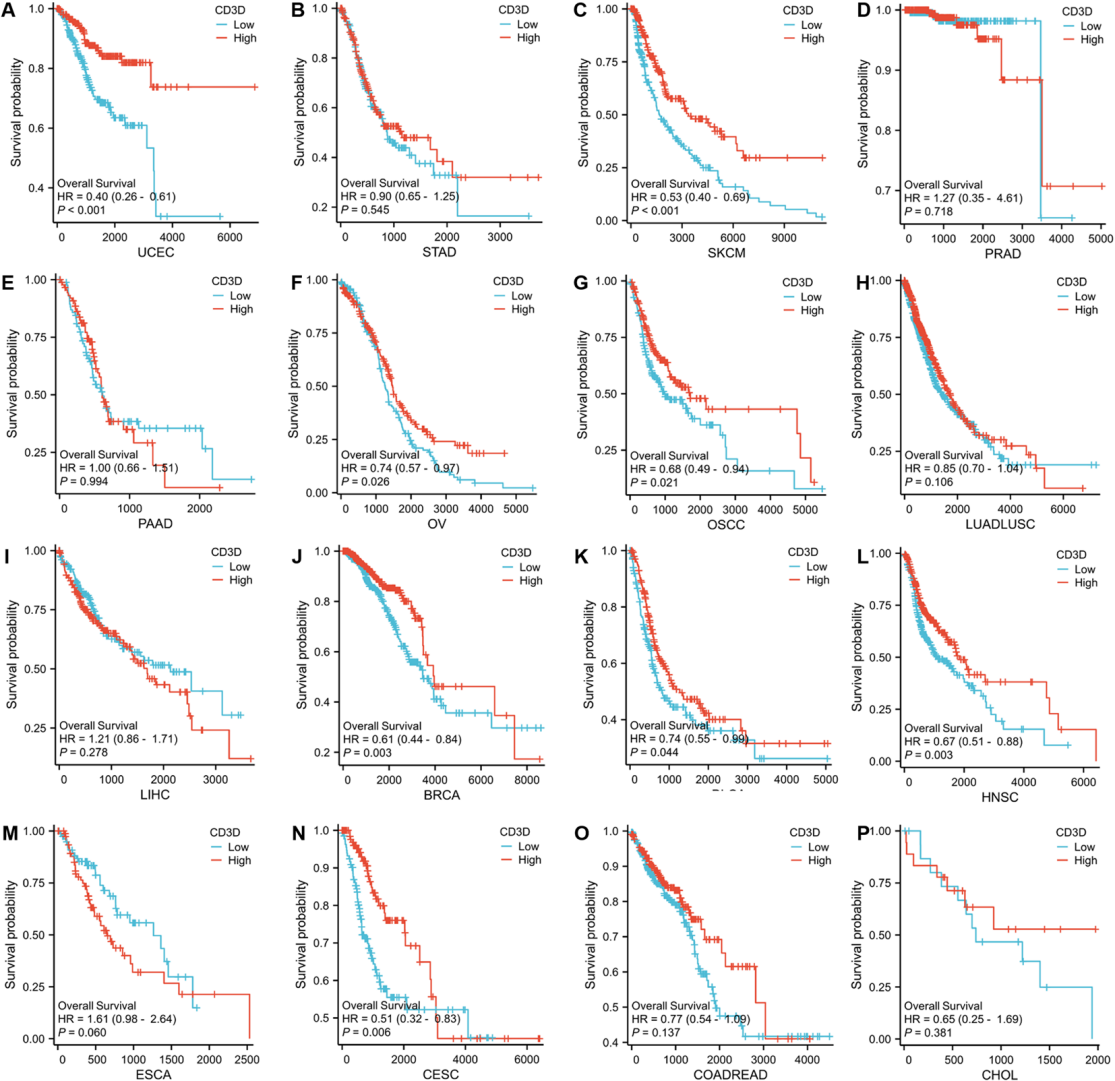
Prognostic Kaplan–Meier curve for CD3D across 16 types of malignant tumors from TCGA data.

Prognostic analysis of 16 common malignant tumor types in the TCGA database demonstrated significant correlations between the expressions of CD3D, CD3E, and CD3G and cancers such as uterine endometrial cancer, ovarian cancer, CC, cutaneous malignant melanoma, breast cancer, and head and neck squamous cell carcinoma (*p* < 0.05) (Fig. 3). PPI analysis via the STRING database identified CD3E, CD3G, CD2, CD4, CD8A, CD247, LCK, TRAT1, and ZAP70 as closely related genes to CD3D.

**Fig. 4:**
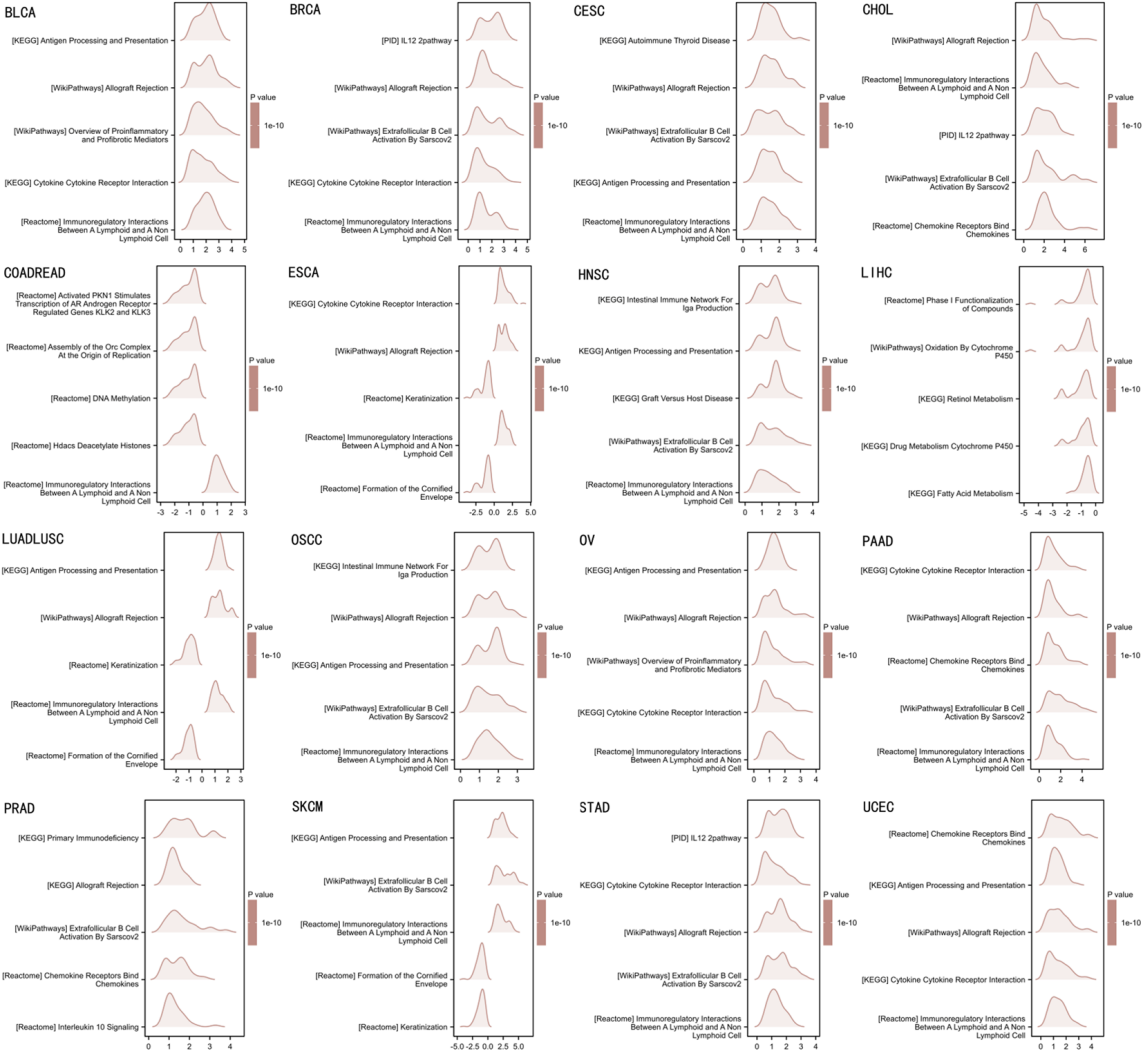
GSEA of genes associated with CD3D gene overexpression.

GSEA, categorizing genes into high- and low-expression groups based on CD3D levels, revealed significant enrichment of immune-related pathways associated with CD3D across all tumor types (Fig. 4).

**Fig. 5:**
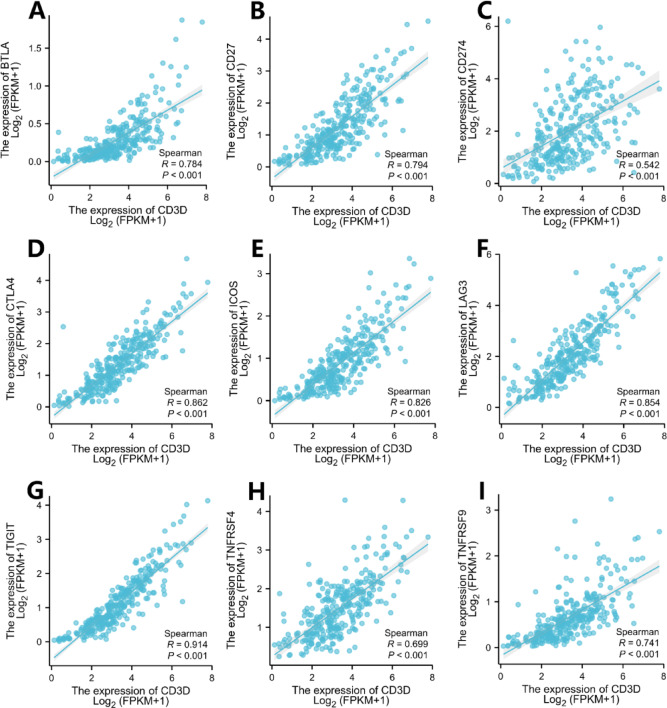
Correlation analysis between immune checkpoints and the CD3D molecule.

Correlation analysis of CD3D expression with immune checkpoint molecules—including BTLA, CD27, CD274, CTLA4, ICOS, LAG3, TIGIT, TNFRSF4, and TNFRSF9—showed strong positive correlations with CD3D, CD3E, and CD3G (Fig. 5). These results highlight the potential of immune checkpoint inhibitors to modulate T lymphocyte populations and shape the anti-cancer immune microenvironment.

### Baseline characteristics of the clinical cohort

The study included 202 patients with early-stage CC, consisting of 119 with low CD3 expression and 83 with high CD3 expression. Patient ages ranged from 26 to 77 years (mean age: 52.3 years). Detailed clinicopathological characteristics are summarized in Table 2.

**Table 2:** Distribution of clinicopathological characteristics in patients with high and low CD3 expression in both the training and test groups.

Characteristics	Training group (n = 140)			Test group (n = 62)		
	High	Low	*p*	High	Low	*p*
Age, years	55.31 ± 9.898	51.582 ± 10.563	0.053	53.976 ± 10.951	52.381 ± 11.061	0.591
Clinical complaint			0.409			0.885
Postcoital bleeding	37 (88.1%)	78 (79.6%)		24 (58.5%)	13 (61.9%)	
Incidental finding	3 (7.1%)	15 (15.3%)		3 (7.3%)	2 (9.5%)	
Increased secretion	2 (4.8%)	5 (5.1%)		14 (34.1%)	6 (28.6%)	
Pregnancy			0.427			0.527
None	0 (0.0%)	1 (1.0%)		0 (0.0%)	0 (0.0%)	
Single	1 (2.4%)	7 (7.1%)		4 (9.8%)	4 (19.0%)	
Several	41 (97.6%)	90 (91.8%)		37 (90.2%)	17 (81.0%)	
Parturition			0.362			0.907
None	0 (0.0%)	4 (4.1%)		0 (0.0%)	0 (0.0%)	
Single	12 (28.6%)	31 (31.6%)		15 (36.6%)	8 (38.1%)	
Several	30 (71.4%)	63 (64.3%)		26 (63.4%)	13 (61.9%)	
HPV			0.103			0.35
Negative	15 (35.7%)	22 (22.4%)		4 (9.8%)	0 (0.0%)	
Positive	27 (64.3%)	76 (77.6%)		37 (90.2%)	21 (100.0%)	
SCC, ng/mL	1.82 (1.078–4.643)	1.12 (0.718–3.132)	0.015*	1.37 (0.92–3.32)	1.68 (0.89–3.8)	0.994
CA125, u/mL	14.45 (9.808–16.875)	14.45 (9.123–17.3)	0.553	13.6 (8.67–17.76)	17.61 (10.22–18.86)	0.252
RBC, 10^12^/L	4.135 (3.762–4.433)	4.115 (3.857–4.365)	0.815	4.07 (3.89–4.4)	4.36 (3.96–4.51)	0.096
PLT, 10^9^/L	229 (185.25–271.5)	239.5 (181–283)	0.592	191 (150–245)	258 (211–313)	0.001*
Neutrophil, 10^9^/L	3.15 (2.322–4.228)	2.915 (2.292–3.628)	0.329	2.92 (2.34–3.91)	3.81 (2.9–4.77)	0.071
Lymphocyte, 10^9^/L	1.845 ± 0.497	1.784 ± 0.575	0.552	1.711 ± 0.705	1.835 ± 0.513	0.249
Hemoglobin, g/L	125.5 (118.5–132.75)	122.5 (114–130)	0.192	122 (113–129)	122 (115–135)	0.557
NLR	1.72 (1.075–2.513)	1.614 (1.324–2.286)	0.955	2.057 (1.535–2.482)	2.023 (1.593–2.41)	0.701
PLR	121.254 (94.379–160.55)	139.069 (104.851–174.722)	0.214	127.966 (92.271–167.361)	151.497 (113.194–173.46)	0.13
Pre FIGO stage			0.338			0.001*
IA	2 (3.6%)	0 (0.0%)		0 (0.0%)	0 (0.0%)	
IB	22 (39.3%)	81 (96.4%)		10 (34.5%)	31 (93.9%)	
IIA	32 (57.1%)	3 (3.6%)		19 (65.5%)	2 (6.1%)	
Tumor longest diameter, mm	32.008 ± 8.888	25.931 ± 10.971	0.002*	24.96 ± 10.827	22.617 ± 5.507	0.047*
Stromal ring			0.001*			0.424
Continuous	14 (33.3%)	62 (63.3%)		20 (48.8%)	8 (38.1%)	
Interrupt	28 (66.7%)	36 (36.7%)		21 (51.2%)	13 (61.9%)	
meanADC, mm^2^/s	0.092 (0.084–0.1)	0.094 (0.067–0.129)	0.588	0.09 (0.078–0.105)	0.096 (0.087–0.099)	0.552
medADC, mm^2^/s	0.089 (0.082–0.099)	0.092 (0.066–0.125)	0.995	0.086 (0.077–0.101)	0.09 (0.084–0.098)	0.48

LVSI, lymph-vascular space invasion; RBC, red blood cell; PLT, platelet; NLR, neutrophil/lymphocyte; PLR, platelet/lymphocyte; HPV, human papilloma virus; SCC, squamous cell carcinoma antigen; CA125, cancer antigen 125; FIGO, 2018, International Federation of Gynecology and Obstetrics; ADC, apparent diffusion coefficients; medADC, median ADC; meanADC, mean ADC; SD, standard deviation.

*p* < 0.05. *represents a statistically significant difference.

Within the training cohort, significant differences were observed between low and high CD3 expression groups in terms of the presence of an intact stromal ring and serum SCC levels. Additionally, the distribution of the longest tumor diameter varied significantly between the two groups in both the training (*p* = 0.02) and test (*p* = 0.047) cohorts. No other clinicopathological parameters showed significant differences (*p* > 0.05).

### Feature selection

From T2WI images, ICC analysis yielded 1487, 1466, and 1479 features from ROI_tumor_, ROI_3mm_, and ROI_5mm_, respectively. For ADCmap images, 1467, 1472, and 1487 features were extracted from ROI_tumor_, ROI_3mm_, and ROI_5mm_, respectively. CE-MRI images provided 1480, 1465, and 1484 features from the same regions.

Feature correlation analysis and F-test selection reduced dataset dimensionality, retaining 18 features for the combined ROI_tumor_ + ROI_3mm_ model derived from mpMRI sequences (Supplementary Table S4).

Univariate analysis of clinical and imaging characteristics (Table 3) revealed significant differences in platelet (PLT) count and tumor longest diameter between low and high CD3 expression groups within the training cohort (*p* < 0.05). These variables were included in subsequent multivariate analyses and model development to enhance predictive performance.

**Table 3:** Clinical predictors of cervical cancer: Univariate and multivariate analyses.

Variables	Univariate analyses		Multivariate analyses		Multivariate analyses	
	OR (95% CI)	*p* value	OR (95% CI)	*p* value	OR (95% CI)	*p* value
Age	1.027 (0.999–1.056)	0.055	1.027 (0.996–1.059)	0.087		
Clinical complaint	1.290 (0.872–1.908)	0.202				
Pregnancy	1.089 (0.919–1.290)	0.326				
Parturition	1.188 (0.880–1.604)	0.260				
HPV	0.764 (0.383–1.562)	0.445				
SCC	1.015 (0.965–1.067)	0.572				
CA125	0.971 (0.937–1.006)	0.101				
RBC	0.902 (0.489–1.663)	0.742				
PLT	0.995 (0.991–0.999)	0.018	0.996 (0.992–0.999)	0.037	0.994 (0.987–0.999)	0.034
Neutrophil	1.089 (0.919–1.290)	0.325				
Lymphocyte	0.959 (0.591–1.558)	0.876				
Hemoglobin	1.004 (0.985–1.023)	0.714				
NLR	1.150 (0.936–1.413)	0.185				
PLR	0.996 (0.991–1.001)	0.131				
Pre FIGO stage	35.87 (0.00–97.263)	0.416				
Tumor longest diameter	1.030 (1.002–1.058)	0.035	1.05 (1.006–1.096)	0.025	1.04 (1.002–1.079)	0. 038
Stromal ring	2.56 (1.165–3.640)	0.013	0.993 (0.433–2.276)	0.987	0.965 (0.200–4.655)	0.965
MeanADC	0.114 (0.013–1.004)	0.049	0.994 (0.98–1.009)	0.43		
MedianADC	0.043 (0.004–0.441)	0.008	1.007 (0.992–1.023)	0.346		
Radscore					8.682 (4.027–21.104)	0.00

Chi-square testing revealed significant differences in CD3-high/CD3-low distribution across FIGO sub-stages (χ^2^ = 77.919, *p* < 0.001), with higher CD3 expression observed in High stages (IIA 61.4% vs. IB 38.6% vs. IA 0%) (Supplementary Figure S1B and Table S5). Incorporating FIGO stage into the clinical model minimally affected the prognostic significance of CD3 status (AUC: 0.738 to 0.772, 95% CI 0.693–0.850). Delong’s test showed no significant difference in ROC curve performance between models with and without FIGO stage (*p* = 0.564, 0.685) (Supplementary Table S6 and S7). Due to these findings and potential multicollinearity between FIGO stage and existing covariates, the original model (without FIGO stage) was retained.

### Model construction and evaluation

**Fig. 6:**
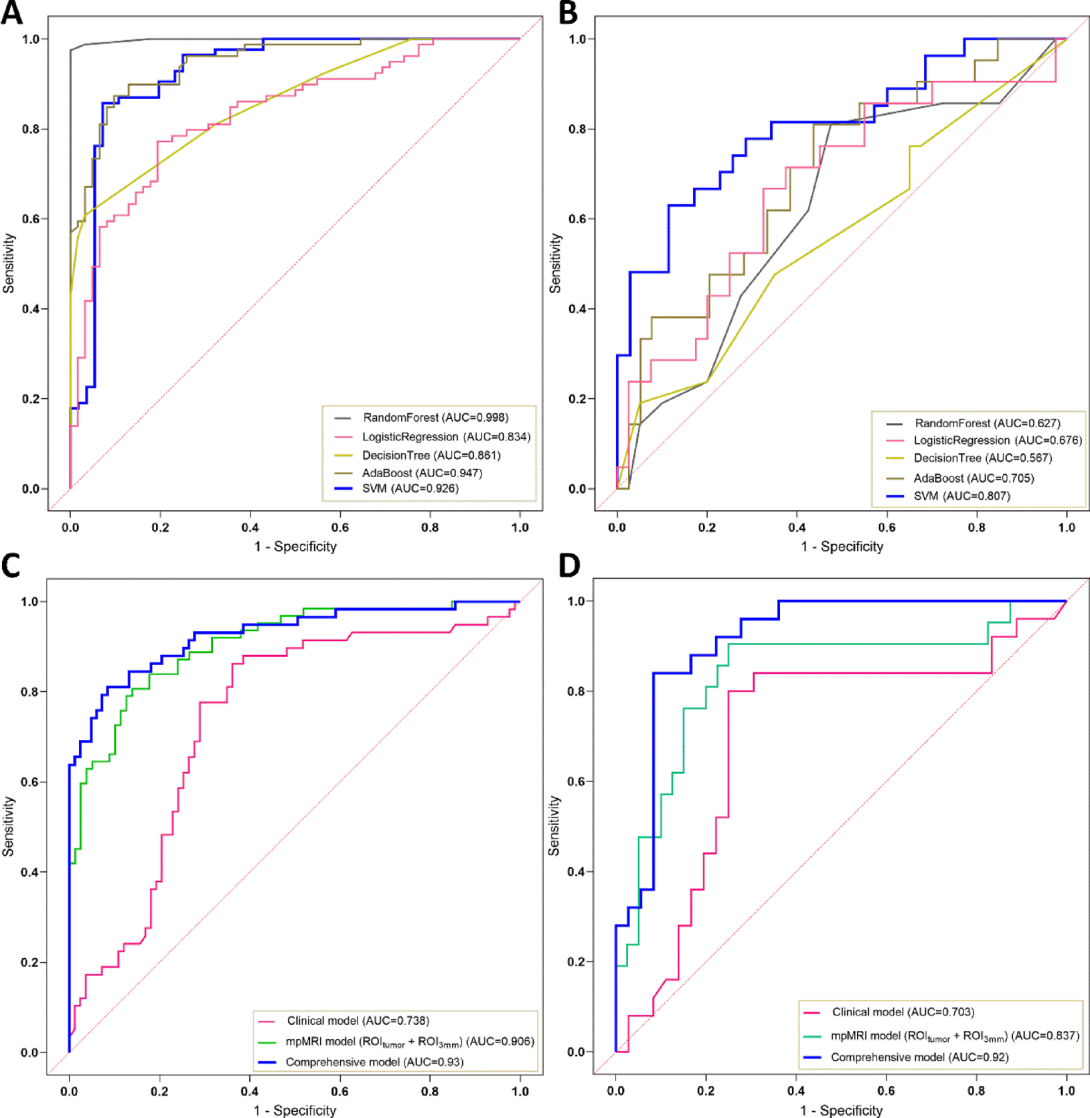
ROC curves for various models. Comparison of ROC curves for five machine learning algorithm models in the training group (**A**) and test group (**B**) based on mpMRI sequences. (**C**) Training group and (**D**) test group analysis for mpMRI ROI_tumor_ + ROI_3mm_, clinical, and integrated models.

The performance of machine learning algorithms utilizing mpMRI data is summarized in Table 4 and visualized in Figs. 6A and B. Five algorithms—SVM, Logistic Regression, Random Forest, AdaBoost, and Decision Tree—were assessed for their predictive capabilities. Among these, the SVM model demonstrated superior predictive performance in both training and test cohorts, as indicated by the ROC curves.

**Table 4:** Performance of various machine learning algorithms in the training and test groups based on mpMRI sequences.

Model	Group	AUC(95%CI)	Accuracy (95% CI)	Sensitivity (95% CI)	Specificity (95% CI)	PPV (95% CI)	NPV (95% CI)
DecisionTree	Training group	0.861 (0.798–0.898)	0.766 (0.695–0.837)	0.968 (0.917–1.000)	0.608 (0.500–0.713)	0.659 (0.560–0.756)	0.96 (0.900–1.000)
	Test group	0.567 (0.516–0.697)	0.459 (0.344–0.574)	0.667 (0.458–0.864)	0.35 (0.212–0.500)	0.35 (0.214–0.500)	0.667 (0.454–0.857)
AdaBoost	Training group	0.947 (0.880–0.979)	0.879 (0.823–0.929)	0.887 (0.800–0.962)	0.873 (0.795–0.939)	0.846 (0.746–0.929)	0.908 (0.842–0.969)
	Test group	0.705 (0.567–0.835)	0.639 (0.508–0.754)	0.714 (0.500–0.895)	0.600 (0.444–0.750)	0.484 (0.313–0.655)	0.800 (0.643–0.933)
RandomForest	Training group	0.998 (0.968–1)	0.979 (0.950–1.000)	0.968 (0.912–1.000)	0.987 (0.96–1.000)	0.984 (0.948–1.000)	0.975 (0.931–1.000)
	Test group	0.627 (0.491–0.773)	0.623 (0.492–0.738)	0.429 (0.211–0.650)	0.725 (0.583–0.861)	0.450 (0.231–0.684)	0.707 (0.548–0.846)
SVM	Training group	0.926 (0.875–0.978)	0.886 (0.829–0.936)	0.929 (0.86–0.984)	0.857 (0.782–0.929)	0.813 (0.708–0.909)	0.947 (0.892–0.988)
	Test group	0.807 (0.696–0.919)	0.774 (0.661–0.871)	0.63 (0.455–0.808)	0.886 (0.763–0.974)	0.810 (0.632–0.957)	0.756 (0.614–0.884)
LogisticRegression	Training group	0.834 (0.783–0.879)	0.780 (0.709–0.851)	0.807 (0.702–0.902)	0.76 (0.663–0.852)	0.725 (0.616–0.822)	0.833 (0.743–0.913)
	Test group	0.676 (0.626–0.706)	0.672 (0.557–0.787)	0.667 (0.476–0.864)	0.675 (0.524–0.829)	0.519 (0.357–0.71)	0.794 (0.657–0.926)

Models incorporating ROI_tumor_, ROI_3mm_, and ROI_5mm_ from CE-MRI, T2WI, and ADC sequences were evaluated across training and test groups (Supplement Table S2). The inclusion of ROI_3mm_ significantly improved predictive efficacy for CD3 expression states compared to models using ROI_5mm_, leading to the selection of ROI_tumor_ and ROI_3mm_ for the final radiomics models to optimize sensitivity and specificity.

Table 5 details the AUC with 95% confidence intervals (CI), accuracy, sensitivity, specificity, PPV, and NPV for the clinical model, radiomics models (ROI_tumor_ + ROI_3mm_ from mpMRI sequences), and the comprehensive model integrating radiomic and clinical data. Figures 6C and D depict the ROC curves for these models in both training and test cohorts. Notably, the comprehensive model, which combines radiomic features with clinical parameters, achieved the highest AUC values, reaching 0.93 (95% CI 0.88–0.97) in the training cohort and 0.92 (95% CI 0.84–0.99) in the test cohort.

**Table 5:** AUC, specificity, sensitivity, accuracy, NPV, and PPV of different models in the training and test groups.

Model	Group	AUC (95% CI)	Accuracy (95% CI)	Sensitivity (95% CI)	Specificity (95% CI)	PPV (95% CI)	NPV (95% CI)
mpMRI (ROI_tumor_ + ROI_3mm_)	Training group	0.906 (0.857–0.954)	0.823 (0.759–0.887)	0.823 (0.722–0.914)	0.823 (0.738, 0.904)	0.785 (0.677–0.881)	0.855 (0.770–0.929)
	Test group	0.837 (0.718–0.956)	0.754 (0.639–0.853)	0.905 (0.750–1.000)	0.675 (0.531–0.816)	0.594 (0.424–0.765)	0.931 (0.828–1.000)
Clinical	Training group	0.738 (0.652–0.823)	0.731 (0.66–0.794)	0.776 (0.662–0.883)	0.699 (0.607–0.788)	0.643 (0.531–0.747)	0.817 (0.723–0.908)
	Test group	0.703 (0.559–0.847)	0.754 (0.639–0.853)	0.840 (0.667–0.963)	0.694 (0.533–0.838)	0.656 (0.471–0.818)	0.862 (0.720–0.969)
Comprehensive	Training group	0.93 (0.88–0.97)	0.865 (0.809–0.922)	0.810 (0.706–0.909)	0.904 (0.838–0.963)	0.855 (0.759–0.943)	0.872 (0.800–0.939)
	Test group	0.92 (0.84–0.99)	0.869 (0.787–0.951)	0.800 (0.650–0.933)	0.917 (0.811–1.000)	0.870 (0.714–1.000)	0.868 (0.75–0.954)

AUC, area under the ROC curve; CI, confidence interval; PPV, positive predictive value; NPV, negative predictive value; mpMRI, CE-MRI + T2WI + ADC sequences; comprehensive model, mpMRI (ROI_tumor_ + ROI_3mm_) + clinical model.

**Fig. 7:**
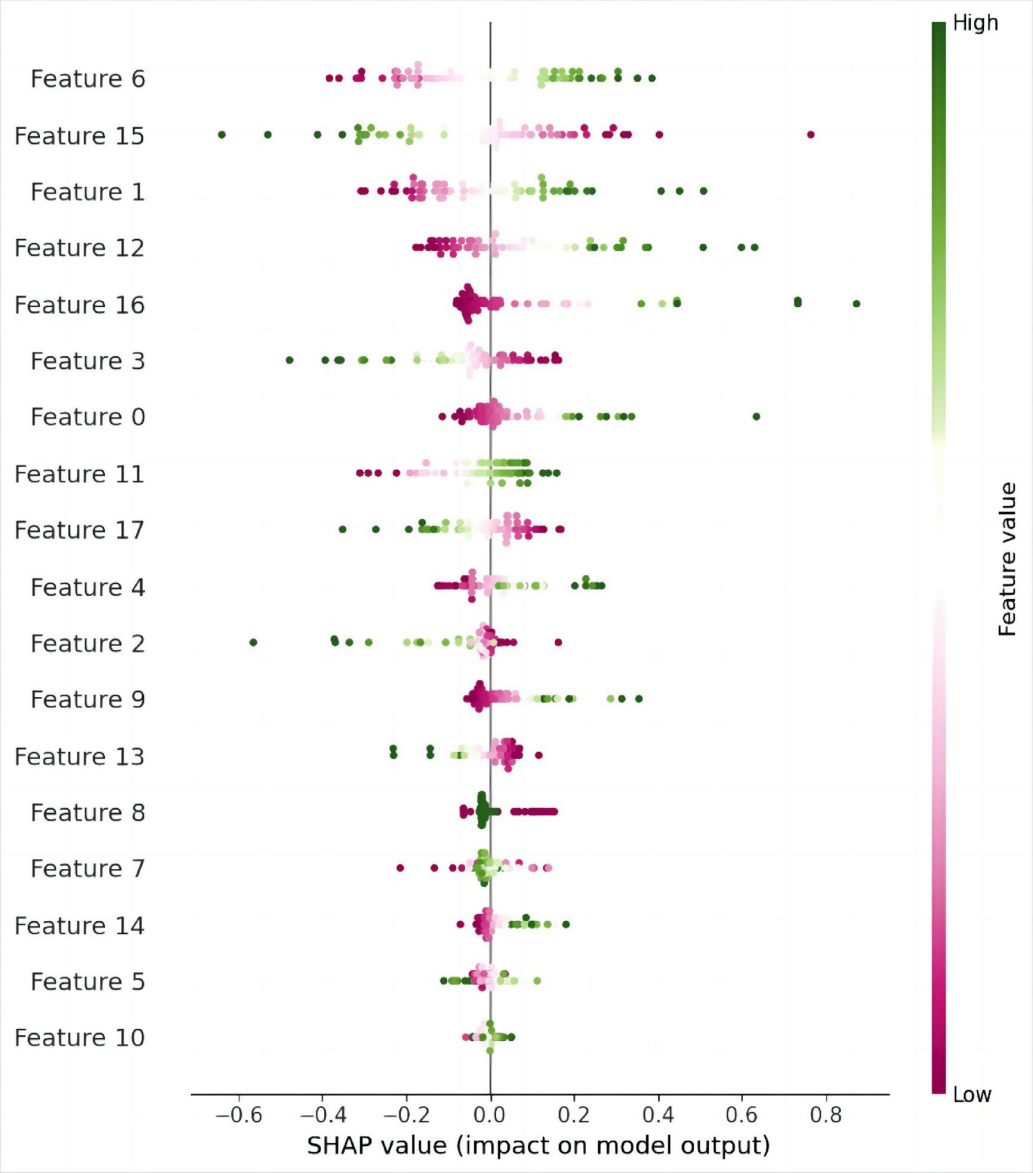
Assessment of the relative influence of model variables in the SVM algorithm, based on SHAP values. Feature0: wavelet-HH_glszm_GrayLevelNonUniformityNormalized_ADC. Feature1: log-sigma-5–0-mm-3D_firstorder_InterquartileRange_ADC. Feature2: lbp-2D_firstorder_Kurtosis_ADC. Feature3: wavelet-HH_glrlm_LongRunEmphasis_ADC. Feature4: wavelet-LH_glcm_Correlation_ADC. Feature5: wavelet-HL_gldm_DependenceVariance_ADC_3mm. Feature6: gradient_glcm_MCC_ADC. Feature7: lbp-2D_glcm_JointEntropy_ADC. Feature8: lbp-2D_firstorder_InterquartileRange_ADC. Feature9: wavelet-LH_gldm_LargeDependenceLowGrayLevelEmphasis_ADC. Feature10: gradient_glcm_Imc2_ADC. Feature11: log-sigma-5–0-mm-3D_glcm_InverseVariance_T1C. Feature12: exponential_firstorder_Minimum_T2W. Feature13: log-sigma-5–0-mm-3D_glcm_DifferenceVariance_T1C. Feature14: logarithm_glszm_HighGrayLevelZoneEmphasis_T2W. Feature15: lbp-2D_firstorder_Skewness_T1C. Feature16: wavelet-HH_ngtdm_Busyness_ADC. Feature17: lbp-2D_gldm_DependenceVariance_T1C.

The influence of model variables was evaluated using SHAP values within the SVM algorithm, as shown in Fig. 7. SHAP analysis identified gradient_glcm_MCC_ADC as the most impactful feature in predicting outcomes, followed by lbp-2D_firstorder_Skewness_T1C, which demonstrated a positive contribution to the model’s predictions. Additional features, such as log-sigma-5-0-mm-3D_firstorder_InterquartileRange_ADC and exponential_firstorder_Minimum_T2W, also contributed significantly, highlighting their importance in the model’s predictive performance.

**Fig. 8:**
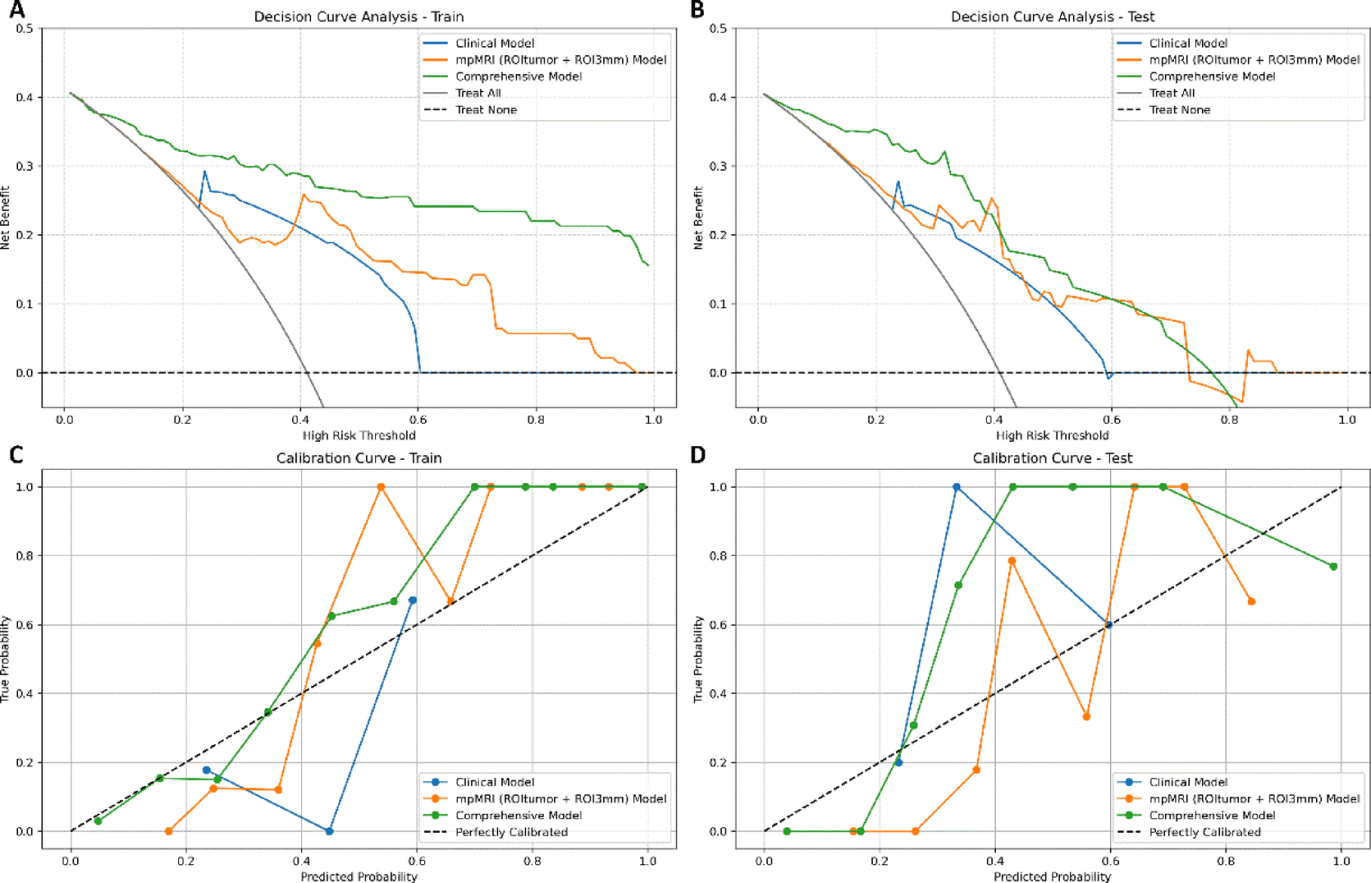
Decision Curve (**A** and **B**) and Calibration curve (**C** and **D**) for the clinical model, radiomics models (ROI_tumor_ + ROI_3mm_ from mpMRI sequences), and integrated model (combination of radiomics and clinical models) in both the training and test groups.

Calibration curve analysis and DCA were conducted for the clinical model, radiomics model (ROI_tumor_ + ROI_3mm_ from mpMRI sequences), and the comprehensive model, indicating excellent calibration and clinical utility in both training and test cohorts (Fig. 8). Calibration curves revealed a strong alignment between predicted probabilities and actual outcomes, with the comprehensive model showing the best agreement, further validating its reliability. DCA demonstrated the clinical utility of the models across a range of threshold probabilities, with the comprehensive model offering the highest net benefit compared to the radiomics model for threshold probabilities between 0 and 0.4. This finding highlights the comprehensive model’s superior capability to balance sensitivity and specificity, underscoring its robustness and practical value in supporting clinical decision-making.

## Discussion

This study developed and validated machine learning models to predict CD3 T cell levels in early-stage CC using radiomic features extracted from tumor regions and 3 mm peritumoral rings on T2WI, ADC, and CE-MRI images. The findings demonstrate a strong association between CD3 complex gene expression and several cancers, including CC, highlighting its potential as a prognostic biomarker. By integrating radiomic features with clinical data, the comprehensive model provides a promising non-invasive method for predicting CD3 expression status. These results underscore the utility of combining radiomics and clinical data to optimize immunotherapy strategies and improve patient outcomes in CC management.

In CC, the extent of T lymphocyte infiltration is a critical indicator of the tumor microenvironment’s immune status and correlates with patient outcomes^10,19–21^. Previous research has shown that higher T lymphocyte levels within the tumor microenvironment enhance cytotoxic activity against tumor cells, thereby improving prognosis^10,11,19^. Consistent with these findings, the current analysis of TCGA data demonstrates that increased T lymphocyte abundance is associated with better prognoses across various cancers, including CC. Routine immunohistochemical assessments have reliably identified T lymphocytes in cancer tissues^12^. TCGA analysis has assisted radiomics in screening biomarkers and revealed the correlation between imaging features and tumor biological behavior. Narang et al.^18^ confirmed that texture features of glioma are associated with CD3 T cell infiltration, with the model’s predictive AUC reaching 0.847. He et al.^17^ constructed a CT radiomics model to predict CTLA4 expression and survival in ccRCC. TCGA analysis may provide a broader molecular basis, such as immune-related gene expression profiles, enhance the integration of radiomics with immunological biomarkers, highlighting the potential of multimodal approaches to improve prognostic accuracy and guide personalized cancer treatment. The combination of these two studies highlights the potential of multimodal approaches. Building on this knowledge, this study focused on identifying MRI-derived imaging biomarkers reflective of T lymphocyte infiltration in CC, aiming to facilitate pre-treatment prediction of immune status and improve treatment planning.

Traditional radiomic studies have primarily focused on analyzing primary tumors, often neglecting peritumoral regions, which play a critical role in the tumor microenvironment^22,23^. Advances in peritumoral radiomics have shown its diagnostic and prognostic potential across various systemic diseases^22–24^. Although definitions of the peritumoral region vary, regions spanning 0 to 10 mm from the tumor boundary—particularly 3 mm and 5 mm—are commonly analyzed^25–28^. In some cancers, the peritumor region may exhibit more pronounced changes in cellular density, necrosis, or structural organization, which could impact the peritumoral optimal distance for radiomic analysis. In this study, radiomic features extracted from 3 and 5 mm peritumoral rings demonstrated that the 3 mm ring provided superior diagnostic accuracy, as reflected by AUC values. These findings align with growing evidence that the immediate peritumoral microenvironment significantly influences tumor-immune interactions and cancer progression.

Radiomic analysis ultimately identified 18 features from each tumor and the 3 mm peritumoral region to construct a predictive model for CD3 expression levels in CC. These features included six wavelet features, four first-order features, three morphological features, and five additional features. SHAP analysis highlighted GLCM features, first-order statistics, and wavelet transform features as the most influential in the model’s predictions. Among these, gradient_glcm_MCC_ADC had the highest contribution, reflecting tumor boundary clarity and internal structural consistency. GLCM plays a crucial role in quantitatively analyzing tumor heterogeneity by reflecting the distribution and positional relationships of pixels, thereby revealing the spatial irregularity within tumors^29,30^. Higher GLCM contrast indicates greater spatial heterogeneity, which is linked to microstructural complexity in tumors. Previous studies have also provided some clues that prognostic value of GLCM-based features in oesophageal cancer^31^. First-order and wavelet-based features captured the biological properties and heterogeneity of the tumors, enhancing the model’s ability to characterize tumor behavior and morphology^32,33^. Although characteristics from all imaging sequences contribute significantly, ADC-based features predominate in our model, which is probably due to their increased sensitivity in identifying minor yet crucial elements of tumor microenvironment. Previous studies have also reported the prognostic value of ADC-based radiomic features in some tumors, underscoring its potential to encapsulate biologically relevant information^15,34^.

The comprehensive model incorporated clinical risk factors, including the longest tumor diameter on axial T2WI images and PLT count. Tumors in the high CD3 expression group tended to be larger, with evidence of deeper stromal or parametrial invasion^35^. This aligns with the 2018 FIGO guidelines, which stress the importance of MRI-based tumor size measurement for accurate staging^34^. Specifically, the longest diameter on axial T2WI was chosen to correspond with transverse stromal penetration depth^36^. Elevated PLT counts, occurring in approximately 10% to 57% of cancer patients, are associated with tumor progression and metastasis through various mechanisms^37,38^. This study identified a negative correlation between elevated PLT counts and CD3 expression levels. This is in agreement with Zhao et al.^39^, who reported that increased PLT counts may act as a poor prognostic indicator for patients with early-stage CC. Similarly, Shi Jia Xin et al.^40^ observed higher PLT counts in patients with CC exhibiting lymph node metastasis compared to those without metastasis (*p* < 0.05) in the training cohort, although this difference was not significant in the validation cohort, aligning with previous reports.

Several limitations warrant consideration. First, the T-lymphocyte analysis relied on public database-derived data, which may be subject to variability in data quality and completeness. Second, the lack of comprehensive survival data and cross-database validation in the CC patient cohort limits the generalizability of the findings. Future studies should aim to develop radiomics models integrating multi-sequence imaging data and clinical parameters from diverse centers. Additionally, the current imaging dataset mainly focuses on early-stage patients and lacks data from advanced stages. Future research plans include increasing the sample size to cover the entire disease course from early to advanced stages, aiming for a more comprehensive perspective and accurate predictive models.

In conclusion, CD3 expression levels correlate significantly with CC staging and prognosis. By integrating intra- and peritumoral multi-sequence MRI radiomic features, tumor size, and PLT counts, this study developed an efficient model for predicting CD3 expression status in preoperative patients with CC. It could aid in identifying patients more likely to benefit from immunotherapy or other immune-targeted treatments, thus informing personalized treatment strategies.

## Electronic supplementary material

Below is the link to the electronic supplementary material.

Supplementary Material 1

Supplementary Material 2

## Data Availability

The datasets generated during the current study are included in this published article. Further inquiries can be available from the corresponding author.

## Declarations

### Study subjects or cohorts overlap

No study subjects or cohorts have been previously reported before.
