# Supplementary material for: Preoperative MRI-based radiomics analysis of intra- and peritumoral regions for predicting CD3 expression in early cervical cancer
Source: Sci Rep. 2025 Jul 23;15:26754. doi: 10.1038/s41598-025-12162-9 (PMC12287529; doi:10.1038/s41598-025-12162-9)
Supplement: Supplementary file 2 — Supplementary Material 2 [file 41598_2025_12162_MOESM2_ESM.docx]

**Figure**


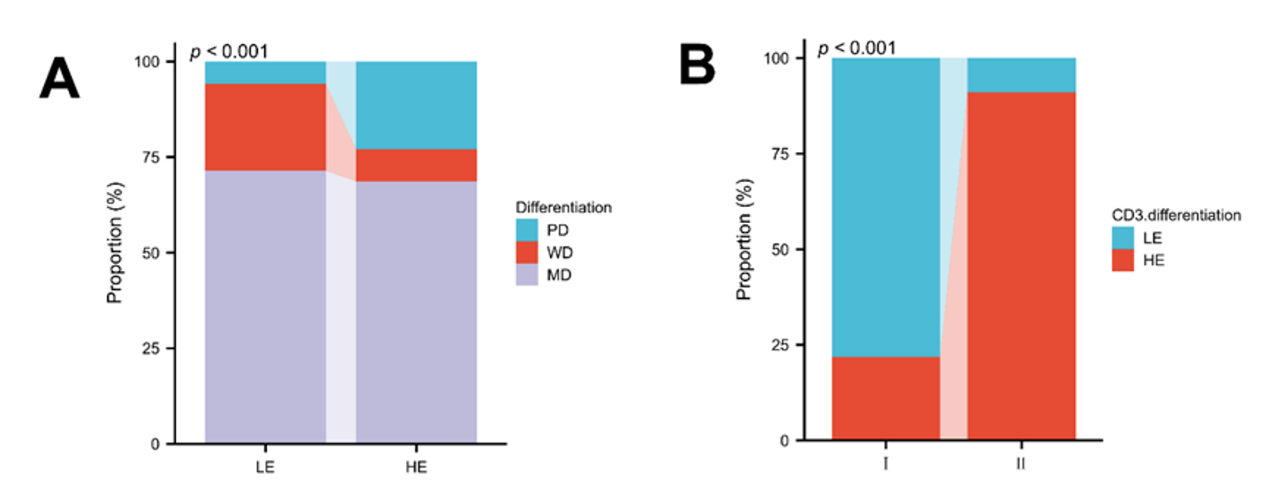


**Figure S1 The correlation analysis of CD3 expression with clinicopathological parameters.**

A. The correlation analysis of CD3 expression with pathological grading. B. The correlation analysis of CD3 expression with FIGO staging. The statistical method was the chi-square test or Fisher's exact test. LE, Low Expression; HE, High Expression; PD, Poor Differentiation; WD, Well Differentiation. MD, Moderate Differentiation. I and II represent FIGO stages.

**Table**

**Table S1. Feature extraction information**

|  | First Order | Shape | GLCM | GLSZM | GLRLM | GLDM | NGTDM | Sum |
| --- | --- | --- | --- | --- | --- | --- | --- | --- |
| original | 18 | 14 | 24 | 16 | 16 | 14 | 5 | 107 |
| wavelet | 72 | 0 | 96 | 64 | 64 | 56 | 20 | 372 |
| LoG | 90 | 0 | 120 | 80 | 80 | 70 | 25 | 465 |
| LBP2D | 18 | 0 | 24 | 16 | 16 | 14 | 5 | 93 |
| Square | 18 | 0 | 24 | 16 | 16 | 14 | 5 | 93 |
| SquareRoot | 18 | 0 | 24 | 16 | 16 | 14 | 5 | 93 |
| Logarithm | 18 | 0 | 24 | 16 | 16 | 14 | 5 | 93 |
| Exponential | 18 | 0 | 24 | 16 | 16 | 14 | 5 | 93 |
| Gradient | 18 | 0 | 24 | 16 | 16 | 14 | 5 | 93 |
| Sum | 288 | 14 | 384 | 256 | 256 | 224 | 80 | 1502 |

GLCM: Gray Level Co-occurrence Matrix, GLDM: Gray Level Dependence Matrix, GLSZM: Gray Level Size Zone Matrix, GLRLM: Gray Level Run Length Matrix, NGTDM: Neighbouring Gray Tone Difference Matrix, LoG: Laplacian of Gaussian, LBP2D: Local Binary Pattern 2D.

**Table S2. hyperparameter of SVM, Logistic Regression, Random Forest, AdaBoost and Decision Tree**

| Model | Hyperparameters |
| --- | --- |
| SVM | C: [0.1, 1, 10, 100]; kernel: ['linear', 'rbf', 'poly']; gamma: ['scale', 'auto']; degree: [3, 4, 5] |
| Logistic Regression | penalty: ['l1', 'l2']; C: [0.01, 0.1, 1, 10, 100]; solver: ['liblinear'] |
| Random Forest | n_estimators: [50, 100, 200]; max_depth: [None, 10, 20, 30]; min_samples_split: [2, 5, 10]; min_samples_leaf: [1, 2, 4] |
| AdaBoost | n_estimators: [50, 100, 200]; learning_rate: [0.01, 0.1, 1.0] |
| Decision Tree | criterion: ['gini', 'entropy']; max_depth: [None, 10, 20, 30]; min_samples_split: [2, 5, 10]; min_samples_leaf: [1, 2, 4] |

**Table S3. Models based on ROItumor, ROI3mm, and ROI5mm from the CE-MRI, T2WI, and ADC sequence** **in the training and test group.**

|  | Models | | | Group | AUC(95%CI) | Accuracy (95%CI) | SEN (95%CI) | SPE (95%CI) | PPV (95%CI) | NPV  (95%CI) |
| --- | --- | --- | --- | --- | --- | --- | --- | --- | --- | --- |
| T2W | ROI_tumor_ | | | Training group | 0.688(0.595-0.781) | 0.643(0.564-0.729) | 0.789(0.674-0.902) | 0.557(0.455- 0.667) | 0.513(0.403-0.63) | 0.817(0.717- 0.913) |
|  |  | | | Test group | 0.639(0.496-0.782) | 0.677(0.565- 0.790) | 0.903(0.774- 1.000) | 0.452(0.28-0.629) | 0.622(0.475- 0.763) | 0.824(0.625- 1.000) |
|  | ROI_tumor_ + ROI_3mm_ | | | Training group | 0.754(0.674-0.835) | 0.638(0.560- 0.709) | 0.944(0.873- 1.000) | 0.448(0.341- 0.545) | 0.515(0.420- 0.611) | 0.929(0.841- 1.000) |
|  |  | | | Test group | 0.732(0.605-0.858) | 0.590(0.475- 0.705) | 0.862(0.727- 0.968) | 0.344(0.176- 0.516) | 0.544(0.396- 0.682) | 0.733(0.467- 0.938) |
|  | ROI_tumor_ + ROI_5mm_ | | | Training group | 0.710(0.622-0.799) | 0.709(0.631- 0.787) | 0.611(0.468- 0.741) | 0.770(0.683- 0.85) | 0.623(0.49-0.75) | 0.761(0.671- 0.852) |
|  |  | | | Test group | 0.686(0.554-0.818) | 0.705(0.59- 0.82) | 0.69(0.519- 0.865) | 0.719(0.552- 0.875) | 0.69(0.517- 0.857) | 0.719(0.556-0.87) |
| ADC | ROI_tumor_ | | | Training group | 0.799(0.720-0.879) | 0.752(0.681- 0.823) | 0.741(0.633- 0.846) | 0.759(0.667-0.847) | 0.683(0.569- 0.79) | 0.808(0.719- 0.886) |
|  |  | | | Test group | 0.701(0.567-0.835) | 0.672(0.557- 0.787) | 0.440(0.240- 0.645) | 0.833(0.703- 0.947) | 0.647(0.412- 0.875) | 0.689(0.537- 0.813) |
|  | ROI_tumor_ + ROI_3mm_ | | | Training group | 0.838(0.774-0.903) | 0.695(0.617- 0.766) | 0.879(0.786- 0.958) | 0.566(0.459- 0.671) | 0.586(0.489- 0.679) | 0.87(0.774-0.959) |
|  |  | | | Test group | 0.777(0.658-0.895) | 0.754(0.639- 0.853) | 0.480(0.3-0.679) | 0.944(0.861- 1.000) | 0.857(0.666- 1.000) | 0.723(0.591- 0.848) |
|  | ROI_tumor_ + ROI_5mm_ | | | Training group | 0.820(0.750-0.891) | 0.759(0.688- 0.83) | 0.69(0.562-0.800) | 0.807(0.725- 0.890) | 0.788(0.706- 0.868) | 0.714(0.6-0.826) |
|  |  | | | Test group | 0.706(0.576-0.835) | 0.623(0.508- 0.754) | 0.840(0.680- 0.963) | 0.472(0.3103- 0.636) | 0.525(0.366- 0.677) | 0.810(0.619- 0.957) |
| T1C | ROI_tumor_ | | | Training group | 0.663(0.572-0.755) | 0.688(0.61- 0.766) | 0.448(0.322- 0.579) | 0.855(0.776- 0.927) | 0.684(0.543- 0.833) | 0.689(0.600- 0.780) |
|  |  | | | Test group | 0.642(0.496-0.788) | 0.656(0.541- 0.771) | 0.48(0.292- 0.692) | 0.778(0.632- 0.903) | 0.600(0.389- 0.818) | 0.683(0.538-0.821) |
|  | ROI_tumor_ + ROI_3mm_ | | | Training group | 0.785(0.709-0.860) | 0.738(0.667- 0.809) | 0.879(0.797- 0.958) | 0.639(0.537- 0.733) | 0.63(0.524- 0.731) | 0.883(0.797- 0.957) |
|  |  | | | Test group | 0.703(0.569-0.838) | 0.689(0.574- 0.803) | 0.720(0.538- 0.893) | 0.667(0.513- 0.824) | 0.600(0.419- 0.788) | 0.774(0.615- 0.909) |
|  | ROI_tumor_ + ROI_5mm_ | | | Training group | 0.761(0.682-0.839) | 0.709(0.624- 0.780) | 0.741(0.627- 0.855) | 0.687(0.578-0.784) | 0.623(0.508- 0.735) | 0.792(0.691- 0.885) |
|  | |  | Test group | | 0.661(0.521-0.801) | 0.607(0.492- 0.721) | 0.840(0.684- 0.963) | 0.444(0.286-0.600) | 0.512(0.361- 0.675) | 0.800(0.625-0.957) |

T2W, T2 weighted; T1C, contrast-enhanced T1-weighted; ADC, apparent diffusion coefficient; ROI, regions of interest; AUC, the area under the curve; SEN, sensitivity; SPE, specificity; PPV, positive predictive value; NPV, negative predictive value.

**Table S4.** **The** **18 features for the combined ROItumor + ROI3mm model derived from the mpMRI sequences.**

| **Number** | **Radiomics features** |
| --- | --- |
| 1 | wavelet-HH_glszm_GrayLevelNonUniformityNormalized_ADC |
| 2 | log-sigma-5-0-mm-3D_firstorder_InterquartileRange_ADC |
| 3 | lbp-2D_firstorder_Kurtosis_ADC |
| 4 | wavelet-HH_glrlm_LongRunEmphasis_ADC |
| 5 | wavelet-LH_glcm_Correlation_ADC |
| 6 | wavelet-HL_gldm_DependenceVariance_ADC_3mm |
| 7 | gradient_glcm_MCC_ADC |
| 8 | lbp-2D_glcm_JointEntropy_ADC |
| 9 | lbp-2D_firstorder_InterquartileRange_ADC |
| 10 | wavelet-LH_gldm_LargeDependenceLowGrayLevelEmphasis_ADC |
| 11 | gradient_glcm_Imc2_ADC |
| 12 | log-sigma-5-0-mm-3D_glcm_InverseVariance_T1C |
| 13 | exponential_firstorder_Minimum_T2W |
| 14 | log-sigma-5-0-mm-3D_glcm_DifferenceVariance_T1C |
| 15 | logarithm_glszm_HighGrayLevelZoneEmphasis_T2W |
| 16 | lbp-2D_firstorder_Skewness_T1C |
| 17 | wavelet-HH_ngtdm_Busyness_ADC |
| 18 | lbp-2D_gldm_DependenceVariance_T1C |

**Table S5. Distribution of CD3-high and CD3-low expression in different subgroups of FIGO IA, IB and IIA.**

| FIGO Substage | High-CD3 Group | Low-CD3 Group | p |
| --- | --- | --- | --- |
| IA | 0 (0%) | 2 (1.7%) | <0.001 |
| IB | 32 (38.6%) | 112 (94.1%) |  |
| IIA | 51 (61.4%) | 5 (4.2%) |  |

**Table S6. AUC, specificity, sensitivity, accuracy, NPV, and PPV of Clinical + FIGO and Clinical models in the training and test groups.**

| Model | Group | AUC(95%CI) | Accuracy | Sensitivity | Specificity | PPV | NPV |
| --- | --- | --- | --- | --- | --- | --- | --- |
| Clinical + FIGO | Training Group | 0.772 (0.693-0.85) | 0.745 | 0.776 | 0.723 | 0.662 | 0.822 |
|  | Test Group | 0.662 (0.521-0.802) | 0.689 | 0.72 | 0.667 | 0.6 | 0.774 |
| Clinical | Training Group | 0.738 (0.652-0.823) | 0.731 | 0.776 | 0.699 | 0.643 | 0.817 |
|  | Test Group | 0.703 (0.559-0.847) | 0.794 | 0.883 | 0.788 | 0.747 | 0.908 |

**Table S7. Comparison of the AUC Values among models**

|  | Training cohort | | |  | Test cohort | | |
| --- | --- | --- | --- | --- | --- | --- | --- |
|  |  | p Value in Comparison to Clinical + FIGO model | p Value in Comparison to clinical model |  |  | p Value in Comparison to Clinical + FIGO model | p Value in Comparison to clinical model |
| Model | *AUC(95% CIs) |  |  |  | AUC(95% CIs) |  |  |
| Clinical + FIGO | 0.772(0.693-0.85) |  | 0.564 |  | 0.662(0.521-0.802) |  | 0.685 |
| Clinical | 0.738(0.652-0.823) | 0.564 |  |  | 0.703(0.559-0.847) | 0.685 |  |
